# 

**Published:** 1919-12-20

**Authors:** 


### Matrons' and Sisters' Appointments.

THE BOROUGH HOSPITAL, BIRKENHEAD.—Miss E. M. Taylor has been appointed sister. She was trained at Brownlow Hill Infirmary, Liverpool, where she was later sister, and she has been sister in the massage, *x*-ray, and electrical department at the General Hospital, Walsall.

WEST HOUSE, ROYAL EDINBURGH ASYLUM, EDINBURGH.—Miss Mary Brabender McGibbon has been appointed night superintendent. She was trained at Gartloch Mental Hospital, and at the Eastern District Hospital, Glasgow, has held the position of sister, and has served in the Q.A.I.M.N.S.R. as sister, night superintendent, and acting matron.

ABERTYSSWG WORKMEN'S HOSPITAL.—Miss Feodora M. Austen has been appointed matron. She was trained at Charing Cross Hospital, has been night sister and theatre sister at the Cancer Hospital, Brompton, and matron of Faringdon Cottage Hospital and of Kington Cottage Hospital.

QUEEN'S HOSPITAL, BIRMINGHAM.—Miss Margaret H. Vincent has been appointed home and tutor sister. She was trained at the Bristol Royal Infirmary and at the Lord Mayor Treloar Hospital, Alton, and she has been ward sister, assistant home sister, and sister-in-charge of the preliminary school at the former institution. Miss Vincent holds the certificate of the Incorporated Society of Trained Masseuses.

UNION INSTITUTION, WOOLWICH ROAD, NEAR GREENWICH.—Miss Maud Lilian Meager has been appointed assistant matron. She was trained at Camberwell Infirmary and at the Soho Hospital for Women; has been staff nurse at South End Military Hospital; sister at the Northampton War Hospital; sister, home sister, night sister, and theatre sister at Bermondsey Infirmary and Military Hospital; deputy holiday matron at the County Tuberculosis Hospital, Ware, Herts; and holiday sister at the West Heath Hospital, Birmingham.

